# Transcription factor-7–like 2 (*TCF7L2*) gene acts downstream of the *Lkb1*/*Stk11* kinase to control mTOR signaling, β cell growth, and insulin secretion

**DOI:** 10.1074/jbc.RA118.003613

**Published:** 2018-07-02

**Authors:** Marie-Sophie Nguyen-Tu, Gabriela da Silva Xavier, Isabelle Leclerc, Guy A. Rutter

**Affiliations:** From the Section of Cell Biology and Functional Genomics and Pancreatic Islet and Diabetes Consortium, Division of Diabetes, Endocrinology and Metabolism, Imperial Centre for Translational and Experimental Medicine, Department of Medicine, Imperial College London, London W12 0NN, United Kingdom

**Keywords:** pancreatic islet, insulin secretion, cell growth, liver kinase B1 (LKB1), T-cell factor (TCF), pancreatic β cell, TCF7L2

## Abstract

Variants in the transcription factor-7–like 2 (*TCF7L2*/*TCF4*) gene, involved in Wnt signaling, are associated with type 2 diabetes. Loss of *Tcf7l2* selectively from the β cell in mice has previously been shown to cause glucose intolerance and to lower β cell mass. Deletion of the tumor suppressor liver kinase B1 (LKB1/STK11) leads to β cell hyperplasia and enhanced glucose-stimulated insulin secretion, providing a convenient genetic model for increased β cell growth and function. The aim of this study was to explore the possibility that *Tcf7l2* may be required for the effects of *Lkb1* deletion on insulin secretion in the mouse β cell. Mice bearing floxed *Lkb1* and/or *Tcf7l2* alleles were bred with knockin mice bearing *Cre* recombinase inserted at the *Ins1* locus (*Ins1Cre*), allowing highly β cell–selective deletion of either or both genes. Oral glucose tolerance was unchanged by the further deletion of a single *Tcf7l2* allele in these cells. By contrast, mice lacking both *Tcf7l2* alleles on this background showed improved oral glucose tolerance and insulin secretion *in vivo* and *in vitro* compared with mice lacking a single *Tcf7l2* allele. Biallelic *Tcf7l2* deletion also enhanced β cell proliferation, increased β cell mass, and caused changes in polarity as revealed by the “rosette-like” arrangement of β cells. *Tcf7l2* deletion also increased signaling by mammalian target of rapamycin (mTOR), augmenting phospho-ribosomal S6 levels. We identified a novel signaling mechanism through which a modifier gene, *Tcf7l2*, lies on a pathway through which LKB1 acts in the β cell to restrict insulin secretion.

## Introduction

Type 2 diabetes currently affects 415 million individuals worldwide, and this number is expected to rise to >600 million by 2040 (www.diabetesatlas.org).^2^ Pancreatic β cell failure is an essential, if still poorly understood, component of disease development and progression (1).

Genome-wide association studies have identified more than 100 loci associated with disease risk (2) with the majority affecting insulin secretion rather than the action of the hormone. Although in a few cases the likely effector transcript has been identified (3, 4), for most loci neither the causal gene nor its mechanism of action at the cellular level has been defined. Of the commonly inherited risk variants, those in the transcription factor-7–like 2 (*TCF7L2*/*TCF4*)^3^ gene, including rs7903146, display among the highest odds ratio for exaggerated type 2 diabetes risk (∼1.2/allele) (5). The identified single-nucleotide polymorphism (SNP) rs7903146 is located in the third intron of TCF7L2 and has been estimated to contribute to 10–25% of all cases of diabetes lean patients (6) TCF7L2 lies at the foot of the wingless (Wnt) signaling pathway activated both by Wnt ligands and by certain growth factors (*e.g.* insulin and IGF-1), which act through receptor tyrosine kinases (7). In the presence of Wnt ligands, a signaling cascade results in stabilization and nuclear localization of β-catenin, which interacts with T cell–specific factor/lymphoid enhancer–binding factor to control transcription of target genes. In the absence of Wnt ligands, β-catenin is degraded by protein complexes, including axin-2 and glycogen synthase kinase 3β (GSK3β) (8).

Several studies have explored the role of *Tcf7l2* in insulin secretion in model systems. Thus, inhibition of TCF7L2 activity in a human or in rat insulinoma cell line (9, 10) inhibited insulin secretion in response to glucose. Likewise, deletion of the *Tcf7l2* gene selectively in the β cell in mice (11, 12) reduced insulin production in older animals and impaired the expansion of β cell mass in response to a high-fat diet (11, 12). Finally, in a separate study (13), re-expression of TCF7L2 on a null background improved glucose tolerance. Importantly, the degree to which the action of disease-risk variants on the β cell may be context-dependent is unclear. Thus, TCF7L2 variants could have different pathophysiological effects among the five different subpopulations of diabetic patients identified in a recent study (14). The mechanisms, including the genetic drivers, behind these differences remain obscure.

Here, we have explored the impact of *Tcf7l2* deletion in a model of β cell expansion driven by artificially enhanced growth factor signaling. Several earlier observations have suggested that a reciprocal relationship may exist between the tumor suppressor liver kinase B1 (LKB1/STK11) and TCF7L2 signaling in other systems. First, the LKB1/STK11 homologue XEEK1 is required for Wnt signaling in *Xenopus laevis* and acts by phosphorylating and inactivating GSK3 (15). Moreover, in Peutz-Jeghers syndrome, Wnt signaling activation is correlated to LKB1 expression (16). Similarly, in esophageal carcinoma patients, LKB1 is down-regulated and Wnt target genes are up-regulated through inhibition of GSK3β activity (17). We (18, 19) and others (20, 21) have shown previously that inactivation of LKB1 in the β cell leads to a substantial increase in insulin production and improved glucose tolerance. LKB1 is a tumor suppressor mutated in Peutz-Jeghers syndrome, a premalignant condition characterized by hamartomatous polyps and an increased risk of all cancers (22, 23). Although the mechanisms involved remain to be fully elucidated, increases in β cell mass (18), changes in the signaling pathways activated by glucose (19, 24), and alterations in cellular morphology and polarity (18, 20, 21) all appear to play a role in enhancing insulin secretion in the *Lkb1*-null β cell. Acting via the fuel-sensitive enzyme AMP-activated protein kinase (AMPK), and the tuberous sclerosis complex TSC1–TSC2, LKB1 also inhibits mammalian target of rapamycin (mTOR) signaling to restrict protein synthesis and cell division (25). This pathway may oppose β cell expansion in the adult because AMPK is likely to be active in these cells in the fasting state (26, 27).

To explore the above possibilities, we used an epistasis approach to examine the impact on the pancreatic β cell of deleting *Tcf7l2* in the absence of *Lkb1* alleles. We show that, in contrast to the action of *Tcf7l2* ablation to impair insulin secretion in WT mice, loss of this transcription factor on an *Lkb1*-null background further increases insulin secretion, β cell size, and β cell mass and augments mTOR activity, consistent with a role for TCF7L2 as an inhibitor of mTOR signaling.

## Results

### Generation of β cell–specific Lkb1/Tcf7l2 double-knockout mice

**Figure 1.**
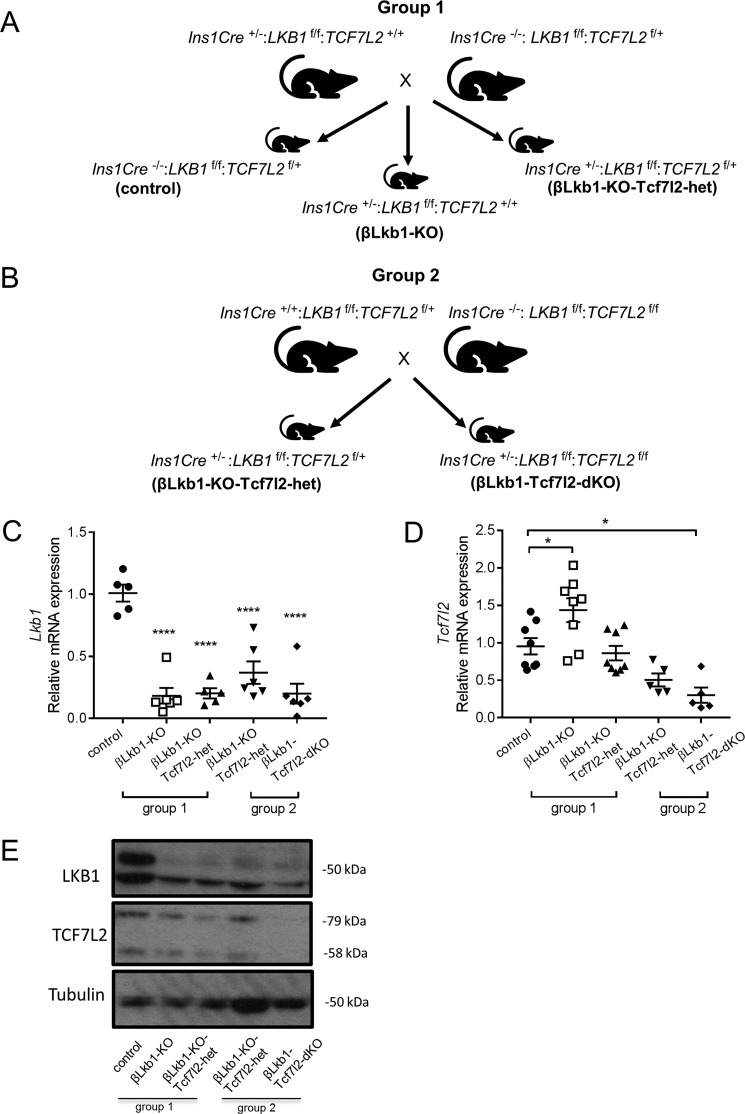
**Breeding strategy for the generation of *Lkb1*/*Tcf7l2* deletion mutants in the β cell and confirmation of the mouse model.** *A*, littermate pups from group 1 display three different genotypes: control as *Ins1Cre*^−/−^*:Lkb1^f^*^/^*^f^:Tcf7l2^f^*^/+^, *Lkb1* deletion only as βLkb1-KO (*Ins1Cre*^+/−^*:Lkb1^f^*^/^*^f^:Tcf7l2*^+/+^), and one single *Tcf7l2* allele deleted in an *Lkb1*-null background as βLkb1-KO-Tcf7l2-het (*Ins1Cre*^+/−^*:Lkb1^f^*^/^*^f^:Tcf7l2^f^*^/+^). *B*, littermate pups from group 2 display two different genotypes; one single *Tcf7l2* allele deleted in an *Lkb1*-null background as βLkb1-KO-Tcf7l2-het (*Ins1Cre*^+/−^*:Lkb1^f^*^/^*^f^:Tcf7l2^f^*^/+^) and two single *Tcf7l2* alleles deleted in an *Lkb1*-null background as βLkb1-Tcf7l2-dKO (*Ins1Cre*^+/−^*:Lkb1^f^*^/^*^f^:Tcf7l2^f^*^/^*^f^*). *C*, RT-qPCR expression of *Lkb1* mRNA in isolated islets (*n* = 5–6 mice/genotype). *D*, RT-qPCR expression of *Tcf7l2* mRNA in isolated islets (*n* = 5–9 mice/genotype). *E*, Western blotting for TCF7L2 and LKB1 in isolated islets (*n* = 2 mice/genotype). *Error bars* represent the mean ±S.E.; *, *p* < 0.05; ****, *p* < 0.0001.

To study the impact of the deletion of *Tcf7l2* and *Lkb1* in the pancreatic β cell, we established breeding pairs on a mixed background (C57BL/6J, FVB/NJ, and 129sS1/SvlmJ) to produce offspring deleted for *Lkb1* and/or *Tcf7l2* selectively in the β cell using the highly selective *Cre* deleter strain *Ins1Cre* in which *Cre* recombinase is inserted into the *Ins1* locus (28, 29) (Fig. 1, *A* and *B*). Deletion at other sites, including the brain, is minimal in this model, and, importantly, the transgene does not carry the human growth hormone minigene present in alternative *Cre* strains (*e.g.* RIP2.Cre) (30). Consequently, effects of *Ins1Cre* expression alone on glucose homeostasis are not observed. Because a strategy generating all possible genotypes would have produced mice homozygous for deletion of both alleles at a frequency of 1 per 64 pups, we designed instead two separate breeding colonies to reduce animal numbers in accordance with the 3Rs. The following offspring were produced and named as follows (group 1): control (*Ins1Cre*^−/−^*:Lkb1^f^*^/^*^f^:Tcf7l2^f^*^/+^),βLkb1-KO (*Ins1Cre*^+/−^*:Lkb1^f^*^/^*^f^:Tcf7l2*^+/+^), and βLkb1-KO-Tcf7l2-het (*Ins1Cre*^+/−^*:Lkb1^f^*^/^*^f^:Tcf7l2^f^*^/+^; Fig. 1*A*, group 1). The second breeding strategy (group 2) generated littermates βLkb1-KO-Tcf7l2-het (*Ins1Cre*^+/−^*:Lkb1^f^*^/^*^f^:Tcf7l2^f^*^/+^) and βLkb1-Tcf7l2-dKO (*Ins1Cre* ^+/−^*:Lkb1^f^*^/^*^f^:Tcf7l2^f^*^/^*^f^*; Fig. 1*B*, group 2).

We first measured *Lkb1* and *Tcf7l2* gene expression in isolated islets using RT-qPCR analysis. The level of endogenous *Lkb1* mRNA was strongly decreased in the presence of *Cre* transgene when one single or both *Tcf7l2* alleles were floxed as expected. Likewise, the level of *Tcf7l2* mRNA was decreased when both alleles were floxed (βLkb1-Tcf7l2-dKO) compared with control. Importantly, we observed no significant differences in the level of *Lkb1* and *Tcf7l2* mRNAs between βLkb1-KO-Tcf7l2-het mice from either group 1 or 2. Of note, deletion of *Lkb1* significantly increased *Tcf7l2* expression when both *Tcf7l2* alleles were present in βLkb1-KO mice (Fig. 1, *C* and *D*). Likewise, we observed decreased LKB1 and TCF7L2 protein expression in isolated islets from βLkb1-KO, βLkb1-KO-Tcf7l2-het, and βLkb1-Tcf7l2-dKO compared with control islets (Fig. 1*E*).

### Deletion in the β cell of two Tcf7l2 alleles in an Lkb1-null background improves oral glucose tolerance and insulin secretion

**Figure 2.**
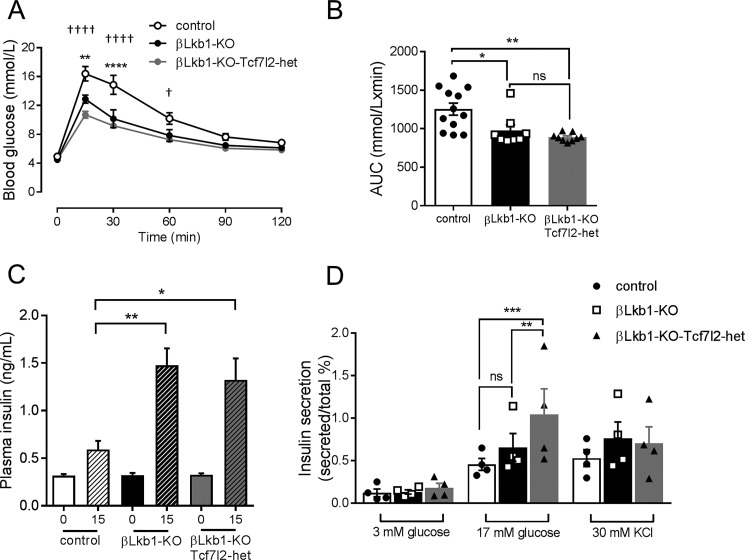
**Monoallelic deletion does not affect *Lkb1* deletion-mediated improvements in glucose tolerance and augmented insulin secretion in males.** *A*, oral glucose (2 g/kg) tolerance measurements were performed as described under “Experimental procedures” (*n* = 9–12 mice/genotype). *p* values were statistically determined by a two-way ANOVA test with a Bonferroni post-test (**, *p* < 0.01 and ****, *p* < 0.0001 βLkb1-KO *versus* control; †, *p* < 0.05 and ††††, *p* < 0.0001 βLkb1-KO-Tcf7l2-het *versus* control; *p* = 0.623 βLkb1-KO *versus* βLkb1-KO-Tcf7l2-het). *B*, area under the curve (*AUC*) for oral glucose tolerance tests (*, *p* < 0.05 βLkb1-KO *versus* control; **, *p* < 0.01 βLkb1-KO-Tcf7l2-het *versus* control). *C*, insulin plasma levels were measured *in vivo* 15 min after intraperitoneal injection of glucose (3 g/kg) (*n* = 7–9 mice/genotype; *, *p* < 0.05 and **, *p* < 0.01 *versus* 15-min control). *D*, insulin secretion *in vitro* was measured from groups of 10 size-matched isolated islets during static incubation (see “Experimental procedures”) and at the indicated glucose or KCl concentrations (*n* = 4 independent experiments; **, *p* < 0.01 βLkb1-KO-Tcf7l2-het *versus* βLkb1-KO; ***, *p* < 0.001 βLkb1-KO-Tcf7l2-het *versus* control). *Error bars* represent the mean ±S.E.; *ns*, not significant.

Consistent with previous findings (18–21, 28), deletion of both *Lkb1* alleles in the β cell improved glucose tolerance in mice aged 8 weeks (Fig. 2, *A* and *B*). These changes were not associated with any alteration in body weight (Fig. S1*A*). Glucose tolerance was not further affected by the additional deletion of a single *Tcf7l2* allele (Fig. 2, *A* and *B*). Deletion of *Lkb1* alone also lowered fed glycemia, and this action was attenuated by the additional deletion of *Tcf7l2* (Fig. S1*B*). Insulin sensitivity was unchanged by deletion of a single *Tcf7l2* allele (Fig. S1*D*).

**Figure 3.**
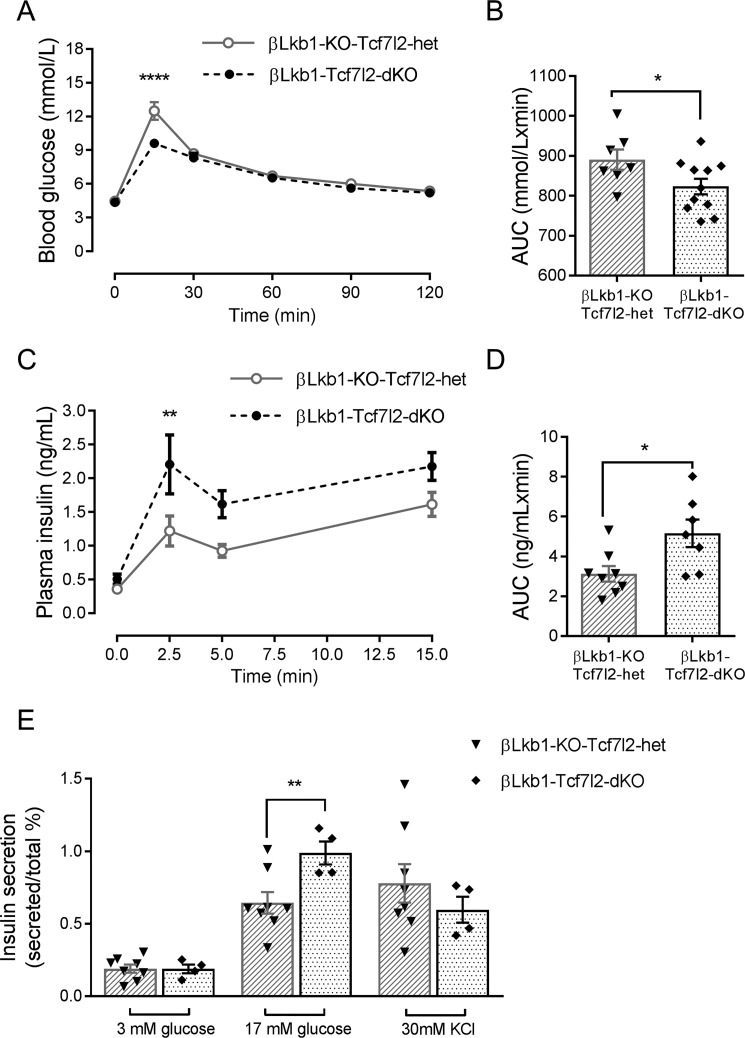
**Deletion of both *Tcf7l2* alleles potentiates the effects of *Lkb1* deletion on glucose tolerance and insulin secretion in males.** Oral glucose tolerance experiments were performed as described in Fig. 2 legend (*A* and *B*) (*n* = 7–11 mice/genotype). Insulin plasma levels were measured *in vivo* 2.5, 5, and 15 min after intraperitoneal injection of glucose (3 g/kg) (*C* and *D*) (*n* = 7–8 mice/genotype). *E*, insulin secretion *in vitro* was measured from groups of 10 size-matched isolated islets during static incubation (see “Experimental procedures”) and at the indicated glucose or KCl concentrations (*n* = 4–6 mice/genotype; *, *p* < 0.05; **, *p* < 0.01; ****, *p* < 0.0001). *Gray open circles*, βLkb1-KO-Tcf7l2-het; *black filled circles*, βLkb1-Tcf7l2-dKO. *Error bars* represent the mean ±S.E. *AUC*, area under the curve.

As described previously (18–21, 28), *Lkb1* deletion substantially increased insulin release in response to glucose *in vivo* (Fig. 2*C*). Interestingly, glucose-stimulated insulin secretion *in vitro* only tended to increase on an *Lkb1*-null background compared with control (Fig. 2*D*). Monoallelic *Tcf7l2* deletion had little further impact on these changes such that the glycemic phenotype of βLkb1-KO-Tcf7l2-het did not differ from βLkb1-KO mice *in vivo*, but insulin release was enhanced *in vitro* (Fig. 2, *C* and *D*). In contrast, when βLkb1-KO-Tcf7l2-het mice were compared with homozygous βLkb1-Tcf7l2-dKO animals deleted for both *Tcf7l2* alleles, we observed a further improvement in glucose tolerance (Fig. 3, *A* and *B*) but unchanged, body weight, fed glycemia, and insulin sensitivity (Fig. S2, *A*, *C*, and *E*). A substantial (∼2-fold) increase in acute insulin release in response to glucose injection was also observed *in vivo* (Fig. 3, *C* and *D*) when comparing βLkb1-Tcf7l2-dKO with βLkb1-KO-Tcf7l2-het littermates. Likewise, comparing islets isolated from mice deleted for both *versus* a single *Tcf7l2* allele, insulin secretion was significantly increased in response to elevated glucose but not to depolarization with KCl (Fig. 3*E*). Thus, deletion of *Tcf7l2* on an *Lkb1*-null background exerts an effect whose direction is opposite to that seen in control islets (11, 12). In females, deletion of one or two *Tcf7l2* alleles on an *Lkb1*-null background did not affect oral glucose tolerance, insulin sensitivity, fed glycemia, and body weight compared with *Lkb1* deletion only (βLkb1-KO; Fig. S2).

**Figure 4.**
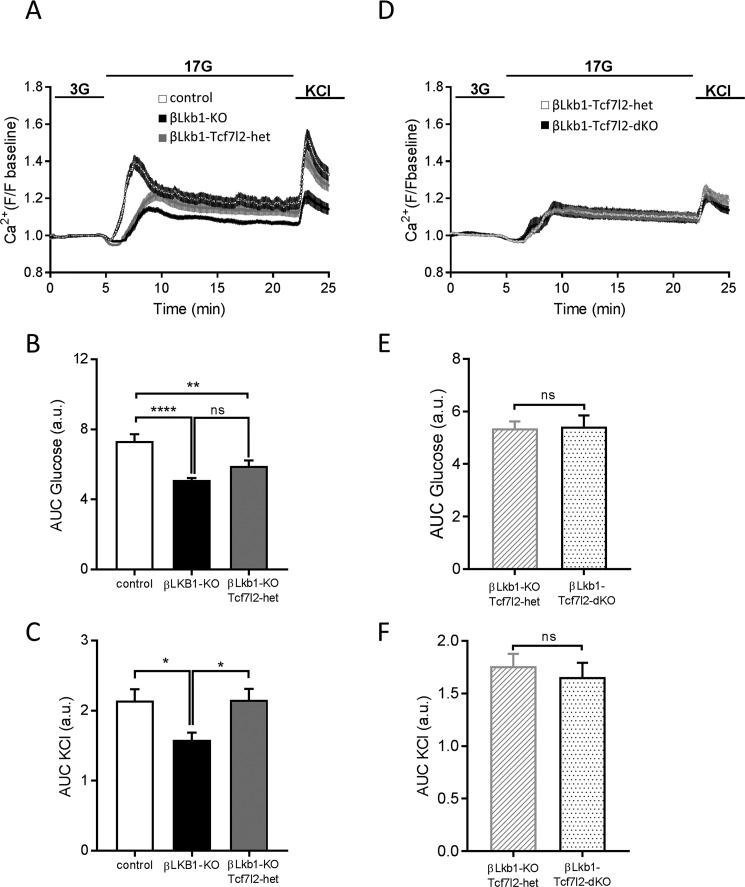
**Deletion of one or two *Tcf7l2* alleles did not further alter Ca^2+^ dynamics in response to glucose but restored responses to KCl in males.** *A*, changes in free cytosolic Ca^2+^ in response to 3 mmol/liter glucose (*3G*), 17 mmol/liter glucose (*17G*), and 20 mmol/liter KCl in group 1. *B*, quantification of area under the curve (*AUC*) for glucose responses. *C*, quantification of area under the curve for KCl responses. *D*, free cytosolic Ca^2+^ changes in response to 3 mmol/liter glucose (*3G*), 17 mmol/liter glucose (*17G*), and 20 mmol/liter KCl in group 2. *E*, quantification of area under the curve for glucose responses. *F*, quantification of area under the curve for KCl responses. Each plot represents the average of 16–29 islets (*n* = 3 per genotype; *, *p* < 0.05; **, *p* < 0.01; ****, *p* < 0.0001). *Error bars* represent the mean ±S.E.; *ns*, not significant. *a.u.*, arbitrary unit.

Next, we sought to explore intracellular free calcium (Ca^2+^) dynamics to elucidate whether these may be altered and contribute to the enhanced insulin secretion. Islets derived from βLkb1-KO mice displayed a delayed and decreased response to high glucose in free cytosolic Ca^2+^ increases compared with control animals (Fig. 4, *A* and *B*). A similar degree of impairment was observed after the additional deletion of a single *Tcf7l2* allele. Interestingly, islets from βLkb1-KO mice showed a decreased response to depolarization with KCl compared with control mice, whereas βLkb1-KO-Tcf7l2-het islets displayed a similar response to KCl compared with control islets (Fig. 4, *A* and *C*). In group 2, no difference in response to high glucose or KCl was noted between islets from βLkb1-KO-Tcf7l2-het and βLkb1-Tcf7l2-dKO mice (Fig. 4, *D*, *E*, and *F*).

### Impact of Lkb1 and Tcf7l2 deletion on islet morphology

**Figure 5.**
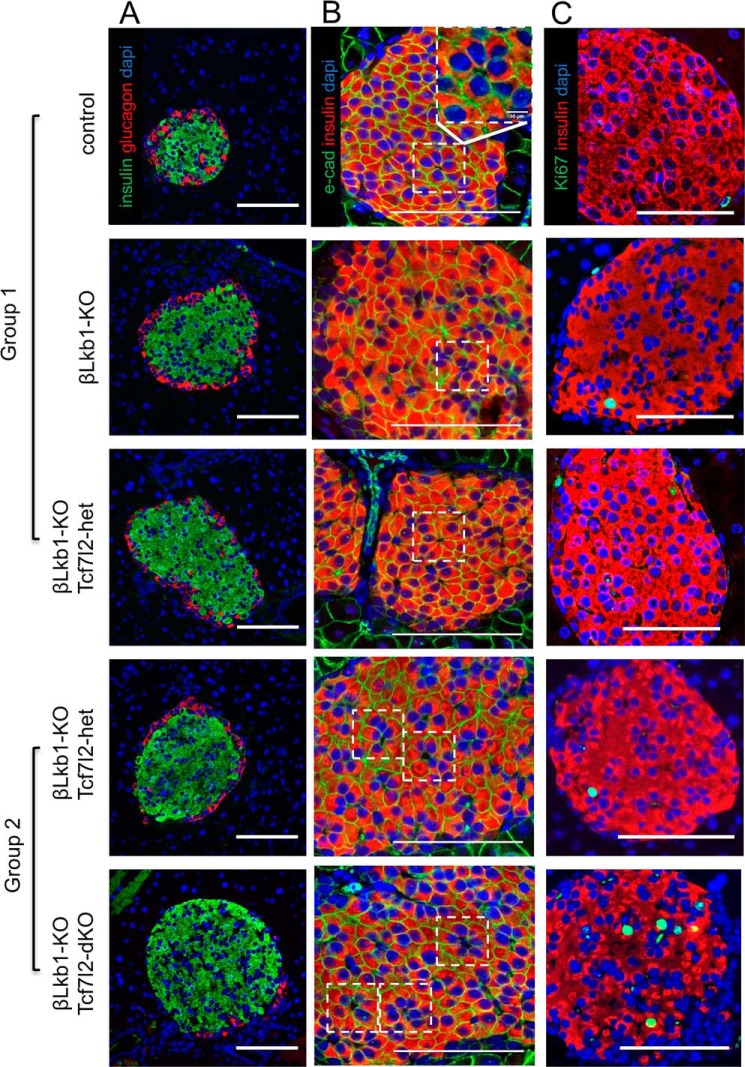
**Impact of *Lkb1* or *Tcf7l2* deletion on islet topography in males.** Representative immunohistochemistry results of pancreatic sections stained for β cell mass (*A*; insulin, 1:200, *green*; glucagon, 1:1000, *red*), an adherens junction marker (*B*; E-cadherin, 1:100, *green*), and a proliferation marker (*C*; Ki-67, 1:100, *green*) are shown. Rosette-like structures are localized in the *white dotted-line square*, and a representative image of an enlarged rosette-like structure is shown in *column B* in control mouse. *Scale bars*, 100 μm.

**Figure 6.**
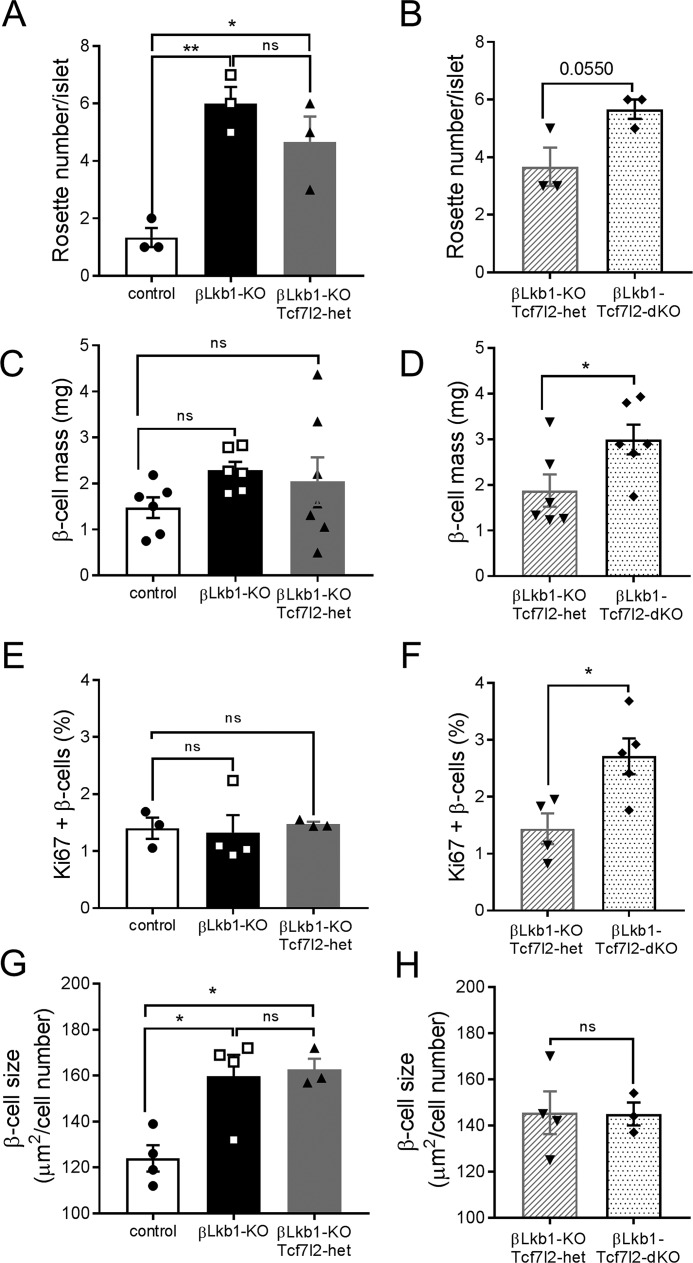
**Effects of *Lkb1* or *Tcf7l2* deletion on β cell size and mass in males.** *A* and *B*, rosette-like structure count per islet (*n* = 3 mice/genotype) in group 1 (*A*) and in group 2 (*B*). *C* and *D*, β cell mass is the ratio of insulin-positive staining to the total pancreatic surface and pancreas weight (*n* = 6–7 mice/genotype). *E* and *F*, quantification of Ki-67–positive and insulin-positive cells based on 10–15 islets per pancreas (*n* = 3–4 mice/genotype). *G* and *H*, mean β cell size measured as the ratio of the insulin-positive staining surface area to the number of β cells (*n* = 3–4 mice/genotype) in group 1 (*G*) and in group 2 (*H*) (*, *p* < 0.05; **, *p* < 0.01). *White bars*, control; *black bars*, βLkb1-KO; *gray bars*, βLkb1-KO-Tcf7l2-het (group 1); *gray hatched bars*, βLkb1-KO-Tcf7l2-het (group 2); *black dotted bars*, βLkb1-Tcf7l2-dKO. *Error bars* represent the mean ±S.E.; *ns*, not significant.

We next examined β cell size and the distribution of β cells within the islet in pancreatic slices (Fig. 5, *A* and *B*). Cellular proliferation was also assessed through Ki-67 staining (Fig. 5*C*). As reported previously (18–21, 28), deletion of *Lkb1* increased the number of “rosette-like” structures within each islet, as identified using the adherens junction marker E-cadherin, likely reflecting a change in cellular polarity (Fig. 6, *A* and *B*) (see (18, 21). The number of rosette structures was not significantly affected by deletion of a single *Tcf7l2* allele, whereas the deletion of both alleles tended (*p* = 0.055) to increase this number, a change that may also contribute to the enhanced secretion observed (18, 21).

β cell mass did not show any significant differences after *Lkb1* deletion, and deletion of a single *Tcf7l2* allele had no further effect (Fig. 6*C*). In contrast, deletion of both *Tcf7l2* alleles caused a substantial (>30%) and significant increase in β cell mass as examined in βLkb1-Tcf7l2-dKO *versus* βLkb1-KO-Tcf7l2-het littermates. Correspondingly, β cell proliferation, examined by Ki-67 staining, was not affected by *Lkb1* deletion alone or the loss of a single *Tcf7l2* allele but significantly increased when two *Tcf7l2* alleles were deleted (Figs. 5*C* and 6, *E* and *F*). β cell size, as assessed by comparing islet volume with the number of DAPI-labeled nuclei/islet, was significantly increased by *Lkb1* deletion but not further affected by either mono- or biallelic deletion of *Tcf7l2* (Fig. 6, *G* and *H*).

### Impact of Tcf7l2 deletion on mTOR signaling

**Figure 7.**
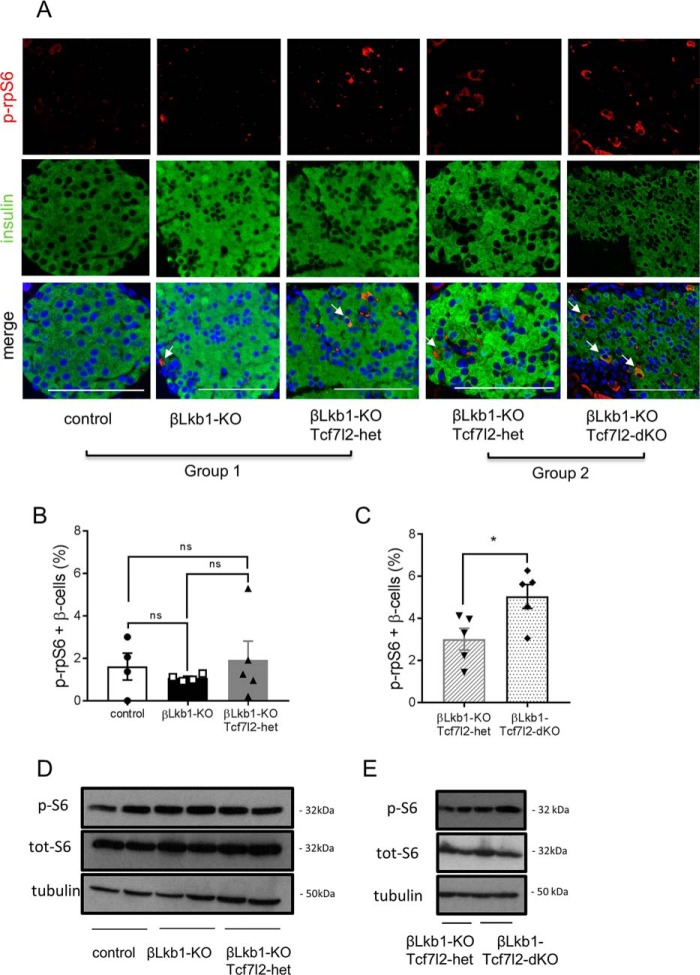
**Deletion of *Tcf7l2* increases mTOR activity in *Lkb1*-null islets from males.** *A*, representative immunofluorescence staining of pancreatic sections from random-fed 10-week-old males using rabbit anti-phospho-ribosomal protein S6 (Ser-235/236) (*p-rpS6*; 1:100; *red*) and guinea pig anti-insulin antibodies (1:200; *red*). *White arrows* represent phospho-rpS6 and insulin colocalization. *Scale bars*, 100 μm. *B* and *C*, quantification of phospho-rpS6–positive staining of total β cells per islet based on 15–20 islets per pancreas from *n* = 4–5 mice/genotype. *White bars*, control; *black bars*, βLkb1-KO; *gray bars*, βLkb1-KO-Tcf7l2-het (*B*), *gray hatched bars*, βLkb1-KO-Tcf7l2-het (*C*); and *black dotted bars*, βLkb1-Tcf7l2-dKO. *D* and *E*, mouse pancreatic islets were isolated from animals of group 1 (*D*) and group 2 (*E*). After an overnight incubation in 11 mmol/liter glucose in RPMI 1640 medium, isolated islets were collected, and lysates from 125 islets were analyzed by immunoblotting with anti-phosphorylated (*p-S6*) and total ribosomal protein S6 (*tot-S6*) (Ser-235/236) and anti-tubulin for group 1 (*D*) and group 2 (*E*). *Error bars* represent the mean ±S.E.; *, *p* < 0.05; *ns*, not significant.

As described previously (18, 20), mTOR signaling is implicated in β cell hypertrophy when *Lkb1* is deleted. We therefore examined whether *Tcf7l2* deletion may impact mTOR signaling. Whereas deletion of *Lkb1* alone had no effect on the levels of phospho-ribosomal protein subunit S6 (rpS6) (Fig. 7, *A*, *B*, and *D*), a significant increase was observed in βLkb1-Tcf7l2-dKO *versus* βLkb1-Tcf7l2-het islets by immunostaining of pancreatic slices (Fig. 7, *A* and *C*) and confirmed by Western (immuno)blotting (Fig. 7*E*).

### Regulation of Wnt signaling

**Figure 8.**
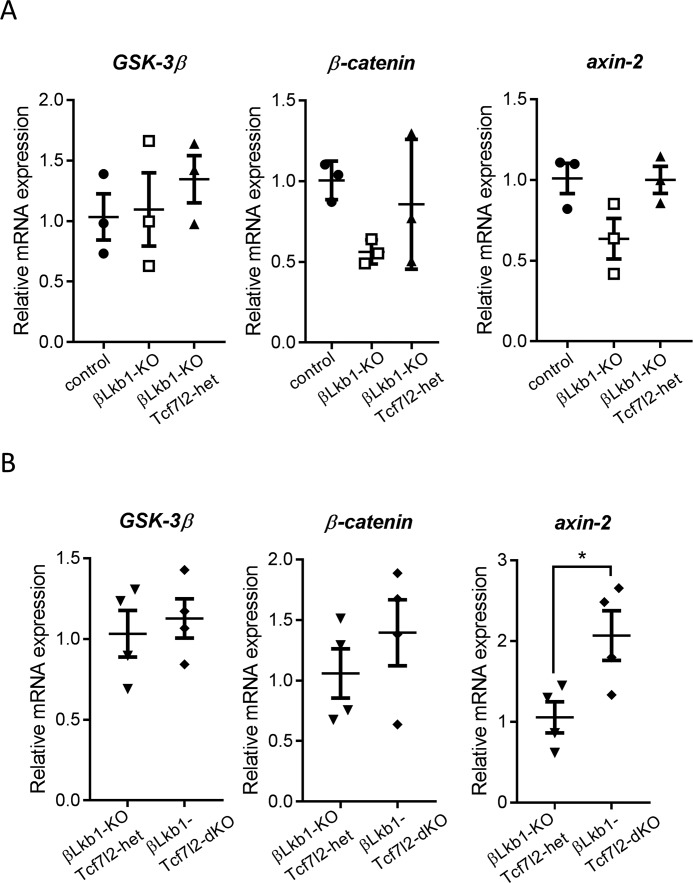
**Wnt signaling is partly dependent on LKB1 and TCF7L2 in males.** Wnt signaling target gene (GSK-3β, β-catenin, and axin-2) expression was assessed by RT-qPCR in isolated islets from group 1 (*A*) and group 2 (*B*) (*n* = 2–3 mice/genotype). *Error bars* represent the mean ±S.E.; *, *p* < 0.05.

Finally, we explored the effects of LKB1 and TCF7L2 deletion on genes in the Wnt/β-catenin pathway. We found that β-catenin, the transcriptional activator for the T cell–specific factor family of transcription factors, and axin-2, a negative loop regulator of Wnt signaling, tend to be down-regulated in the absence of LKB1 (Fig. 8*A*). However, axin-2 was up-regulated by LKB1 and TCF7L2 deletion (Fig. 8*B*). Therefore, it is possible that a cross-talk exists between LKB1 and Wnt/TCF7L2 signaling in pancreatic islets and that this could be involved in controlling β cell proliferation. Furthermore, it is possible that Lkb1 could be a regulator of Wnt signaling and that TCF7L2 could contribute to loop regulation of Wnt signaling involved in proliferative signaling induced by Wnt ligands.

## Discussion

The overall aim of the present study was to determine whether, under conditions of exaggerated β cell proliferation, the role of *Tcf7l2* may differ from that previously described in animals placed under metabolic stress imposed by aging or by a high-fat diet (11, 12). To this end, we used a mouse model in which *Lkb1* was deleted selectively in β cells, mimicking, at least in part, changes during early development (31, 32), pregnancy (33, 34), and insulin resistance (“compensation”) prior to the onset of type 2 diabetes (35, 36). This seemed an important question given that apparent differences in action have previously been described for other genome-wide association study–identified type 2 diabetes genes, such as *SLC30A8* (37–39), when modeled in mice.

Strikingly, we demonstrated that the direction of the effect of *Tcf7l2* deletion is reversed under these conditions (β cell hyperfunction) *versus* those seen under metabolic stress (11, 12). Given that TCF7L2 is normally considered to be a positive regulator of the cell cycle and thus proproliferative (40, 41), this result was unexpected. We therefore considered carefully the possibility that this might be due to alterations elsewhere in the genome given that the *Lkb1* alleles (FVB/N/129S1) were carried by animals with a slightly different genetic background from the floxed *Tcfl72* strain (C57BL/6J) used (see Table S1). Although this possibility cannot be excluded absolutely, we believe it is unlikely given that both FVB/N (42) and 129S1 (43) animals display similar glucose tolerance on a regular chow diet as C57BL/6 mice.

As an alternative explanation, we speculated that *Tcf7l2* acts as a negative regulator of mTOR signaling. This view was supported by the data shown in Fig. 7, which demonstrated increased mTOR signaling after deletion of both, but not a single, *Tcf7l2* allele. Interestingly, in the present study, we saw relatively little effect of *Lkb1* deletion on mTOR signaling in the presence of *Tcf7l2* despite the predicted activation of the downstream TSC1–TSC2 complex in the absence of AMPK activity (25). Nevertheless and interestingly, loss of TCF7L2 impacted the alterations in β cell apical–basolateral polarity observed after *Lkb1* ablation (18–21, 28), which led to alterations in the number of “rosette” structures. The latter changes have previously been ascribed to alterations in signaling by the AMPK-related kinase MARK2/*Par1b* (21).

Interestingly, increased *Tcf7l2* and decreased β-catenin mRNA levels were also observed after *Lkb1* deletion in the present study, providing evidence for an interaction between these genes in the β cell wherein LKB1 represses *Tcf7l2* expression (Fig. 8). Moreover, we found that axin-2 was regulated positively and negatively, respectively, by *Lkb1* and *Tcf7l2*.

We also noted that deletion of *Tcf7l2* on an *Lkb1*-null background resulted in changes in β cell growth (*i.e.* hypertrophy and hyperplasia) but also increased β cell function (secretion of insulin as normalized to total insulin content). Although increased mTOR signaling provides a likely mechanism for the former, the mechanisms driving increased insulin secretion remain unclear. In recent studies, we (19) and others (24) demonstrated that loss of LKB1 signaling resulted in marked alterations in glucose signaling to ATP generation and calcium dynamics such that the so-called “amplifying” pathway of insulin secretion (44, 45), possibly mediated by enhanced synthesis of glutamate and other amino acids, became the predominant means through which hormone release was activated in response to the sugar. The present study confirmed these findings (Fig. 4*A*). Importantly, we observed no evident improvement in Ca^2+^ dynamics in response to elevated glucose or KCl after deletion of a single or both *Tcf7l2* alleles. This observation argues against the view that *Tcf7l2* deletion leads to a reversion to a more conventional route for glucose-stimulated insulin secretion, chiefly reliant on the closure of ATP-dependent K^+^ channels (46) and calcium influx. Instead, the new findings point toward a further enhancement of the amplifying pathways for insulin secretion in β cells lacking both *Tcf7l2* alleles in the absence of *Lkb1*.

The present data may also provide a mechanistic underpinning for other findings in the literature that have pointed to a possible interaction between nutrient levels and *TCF7L2* action. For example, the action of *TCF7L2* risk (T) allele rs7903146 depended on plasma glucose levels during oral glucose tolerance tests (47) with deleterious actions being most apparent at high glucose and a tendency to be protective at low glucose. We would note that, although the direction of this effect might appear to be the reverse of that reported here in mice, the above study chiefly interrogated the actions of incretins on insulin secretion; incretins were not examined here. Nevertheless, glucose-dependent suppression of AMPK activity, likely to mimic the effect of *Lkb1* deletion on mTOR activity, may provide a means through which changes in glycemia modulate the direction of the effect of *TCF7L2* variants on type 2 diabetes risk.

In summary, we demonstrate here that *Tcf7l2* acts as a modifier gene for *Lkb1* in the β cell, affecting islet polarity, cellular proliferation, and mass via mTOR signaling. These findings may be relevant for our understanding of the actions of human *TCF7L2* variants on type 2 diabetes risk in different individuals and settings (14).

## Experimental procedures

### Generation of mutant mice lacking LKB1 and TCF7L2 selectively in pancreatic β cells

Mice homozygous for the floxed *Lkb1*/*Stk11* gene (mixed FVB/129S1 and C57BL/6 background) (18) were crossed to mice homozygous for floxed (f/f) *Tcf7l2* alleles (C57BL/6 background) (12). The resulting double heterozygotes (*Lkb1^f^*^/+^*:Tcf7l2^f^*^/+^) were crossed with double heterozygous mice, and the latter were then bred with mice expressing *Cre* recombinase at the insulin 1 locus (*Ins1Cre*) (28, 29). Subsequently, two separate breeding colonies were established to produce the following offspring and named as follows (group 1): control (*Ins1Cre*^−/−^*:Lkb1^f^*^/^*^f^:Tcf7l2^f^*^/+^), βLkb1-KO (*Ins1Cre*^+/−^*:Lkb1^f^*^/^*^f^:Tcf7l2*^+/+^), and βLkb1-KO-Tcf7l2-het (*Ins1Cre*^+/−^*:Lkb1^f^*^/^*^f^:Tcf7l2^f^*^/+^; Fig. 1*A*). The second breeding strategy generated littermates βLkb1-KO-Tcf7l2-het (*Ins1Cre*^+/−^*:Lkb1^f^*^/^*^f^:Tcf7l2^f^*^/+^) and βLkb1-Tcf7l2-dKO (*Ins1Cre* ^+/−^*:Lkb1^f^*^/^*^f^:Tcf7l2^f^*^/^*^f^*; Fig. 1*B*, group 2). The genetic background of the resulting crosses was quantified by SNP genome scanning analysis (The Jackson Laboratory; Table S1).

### Mouse maintenance and diet

Animals were housed two to five per individually ventilated cage in a pathogen-free facility with 12-h light/dark cycle and had free access to standard mouse chow diet. Unless otherwise stated, data presented are those obtained using male mice. All *in vivo* procedures described were performed at the Imperial College Central Biomedical Service and approved by the UK Home Office Animals Scientific Procedures Act, 1986 (HO License PPL PA03F7F07 to I. L.).

### Measurement of metabolic parameters in vivo

Glucose tolerance was performed on 15-h–fasted mice after an oral gavage of glucose (2 g/kg of body weight). Tail venous blood glucose was monitored at 0, 15, 30, 60, 90, and 120 min after glucose administration. Insulin tolerance was performed on 5-h–fasted mice after an intraperitoneal injection of insulin (0.75 unit/kg of body weight; Humulin® S; Lilly). Tail venous blood glucose was monitored at 0, 15, 30, and 60 min. *In vivo* glucose-stimulated insulin secretion was assessed after intraperitoneal injection of glucose (3 g/kg), and blood was collected at 0, 2.5, 5, and 15 postinjection. Plasma insulin levels were measured using a homogenous time-resolved fluorescence (HTRF) mouse insulin kit (Cisbio, France).

### Isolation of mouse islets

Islets were isolated by digestion with collagenase as described (48). In brief, pancreata were inflated with a collagenase solution (1 mg/ml) and placed in a water bath at 37 °C for 12 min. After several washes, the islets were purified on a Histopaque gradient (Sigma-Aldrich), and isolated islets were cultured for 24 h in RPMI 1640 medium containing 11.1 mm glucose, 10% fetal bovine serum, and l-glutamine (Sigma-Aldrich) and allowed to recover overnight.

### Ex vivo glucose-stimulated insulin secretion

Insulin secretion assays on isolated mouse islets were performed as described previously (18). In brief, 10 size-matched islets per condition were incubated for 1 h in Krebs-HEPES-bicarbonate (KHB) solution (130 mm NaCl, 3.6 mm KCl, 1.5 mm CaCl_2_, 0.5 mm MgSO_4_, 0.5 mm KH_2_PO_4_, 2 mm NaHCO_3_, 10 mm HEPES, and 0.1% (w/v) BSA, pH 7.4) containing 3 mm glucose. Subsequently, islets were incubated for 30 min in KHB solution with either 3 mm glucose, 17 mm glucose, or 30 mm KCl. Secreted insulin and total insulin were quantified using an HTRF insulin kit (Cisbio) in a PHERAstar reader (BMG Labtech, UK) following the manufacturer's guidelines.

### RNA extraction and quantitative real-time PCR analysis

RNA was isolated and purified from fresh isolated islets (50–200) with TRIzol reagent (Invitrogen) according to the manufacturer's instructions. RNA purity and concentration were measured by spectrophotometry (Nanodrop, Thermo Fisher), and only RNA samples with an absorption ratio between 1.8 and 2.0 for 260/280 nm were used. cDNA was synthesized using 200 ng of RNA using the High-capacity cDNA Reverse Transcription kit (Applied Biosystems) including random primers. For quantitative real-time PCR, we used SYBR Green PCR Master Mix (Life Technologies) and the primers sequences in Table S2.

### Immunohistochemistry and islet morphology

Isolated pancreata were removed from euthanized mice, fixed overnight in 10% (v/v) formalin, and subsequently embedded in paraffin wax. Sections (5 μm) were cut and fixed in Superfrost slides. Slides were prepared as detailed previously (28). For antigen retrieval before specific antigen detection, sections were treated with Tris-EDTA buffer, pH 9.0, at 95 °C for 20 min. Primary antibodies used were anti-guinea pig insulin (1:200; Dako), anti-mouse glucagon (1:1000; Sigma-Aldrich), anti-E-cadherin (1:100; Cell Signaling Technology), and anti-Ki-67 (1:200; Abcam, UK). Slides were visualized using an Axiovert 200 M microscope (Zeiss, Germany) with Alexa Fluor 488 goat anti-guinea pig IgG, Alexa Fluor 568 donkey anti-mouse IgG, Alexa Fluor 488 goat anti-rabbit IgG, or Alexa Fluor 568 goat anti-guinea pig IgG (Invitrogen). ImageJ software (Wayne Rasband, National Institute of Mental Health) was used to calculate the β cell mass and size. We determined the percentage of pancreatic surface that was insulin- or glucagon-positive as measured in four sections separated by 75 μm in the *z* axis from six to seven mice of each genotype. For Ki-67 and E-cadherin detection, pancreata from three 10-week-old mice in each genotype were examined. At least three 5-μm sections per mouse at least 150 μm apart were analyzed. To quantify the number of rosette-like structures (*i.e.* 8–10 cells arranged concentrically around an identifiable central “core”) in islets (18), we used E-cadherin and DAPI staining of pancreatic sections. Structures were included where the void at the center was negative for DAPI. Ten islets per mouse and three mice per genotype were assessed.

### Western (immuno)blotting

After isolation, islets were collected and lysed in ice-cold buffer (150 mm NaCl, 10 mm Tris-HCl, pH 7.2, 0.1% SDS, 1% deoxycholate, 5 mm EDTA, and 1% Triton X-100) containing protease inhibitor mixture (Roche) and phosphatase inhibitors (Sigma-Aldrich). Lysates from 125 islets were denatured for 5 min at 95 °C in Laemmli buffer, resolved by 10% SDS-PAGE, and transferred to polyvinylidene difluoride membranes before immunoblotting. Intensities were quantified using ImageJ.

### Antibodies

The following antibodies were used in Western (immuno) blot analysis and immunohistochemistry: rabbit anti-phospho-S6 ribosomal protein (Ser-235/236) (Cell Signaling Technology), mouse anti-α-tubulin (Sigma-Aldrich), rabbit anti-E-cadherin (Cell Signaling Technology), guinea pig anti-insulin (Dako), mouse anti-glucagon (Sigma-Aldrich), and rabbit anti-Ki-67 (Abcam).

### Measurement of intracellular free calcium

Whole isolated islets were incubated with Fura-8AM (Invitrogen) for 45 min at 37 °C in KHB containing 3 mmol/liter glucose. Fluorescence imaging was performed using a Nipkow spinning disk head, allowing rapid scanning of islet areas for prolonged periods of time with minimal phototoxicity. Velocity software (PerkinElmer Life Sciences) provided the interface while islets were kept at 37 °C and constantly perifused with KHB containing 3 or 17 mmol/liter glucose or 20 mmol/liter KCl. For each experiment, we used 16–29 islets. Imaging data were analyzed with ImageJ software using an in-house macro (49).

### Statistical analysis

GraphPad Prism 7.0 was used for statistical analysis. Statistical significance was evaluated by two-tailed paired Student's *t* test and one- or two-way ANOVA with a Bonferroni or Tukey post hoc test when appropriate. All data are shown as means ± S.E. *p* values of <0.05 were considered statistically significant.

## Author contributions

M.-S. N.-T. data curation; M.-S. N.-T. formal analysis; M.-S. N.-T., I. L., and G. A. R. methodology; M.-S. N.-T. and G. A. R. writing-original draft; G. d. S. X. and I. L. writing-review and editing; G. A. R. conceptualization; G. A. R. funding acquisition; G. A. R. investigation.

## Supplementary Material

Supporting Information

## Footnotes

2 Please note that the JBC is not responsible for the long-term archiving and maintenance of this site or any other third party-hosted site.

## Footnotes

3 The abbreviations used are:

TCF7L2: transcription factor-7–like 2
AMPK: AMP-activated protein kinase
GSK3: glycogen synthase kinase 3
LKB1: liver kinase B1
mTOR: mammalian target of rapamycin
TSC: tuberous sclerosis complex
qPCR: quantitative PCR
KO: knockout
dKO: double knockout
DAPI: 4′,6-diamidino-2-phenylindole
rpS6: ribosomal protein subunit S6
HTRF: homogenous time-resolved fluorescence
KHB: Krebs-HEPES-bicarbonate
ANOVA: analysis of variance.
