# Supplementary material for: Transcription factor-7–like 2 (TCF7L2) gene acts downstream of the Lkb1/Stk11 kinase to control mTOR signaling, β cell growth, and insulin secretion
Source: J Biol Chem. 2018 Jul 2;293(36):14178–89. doi: 10.1074/jbc.RA118.003613 (PMC6130960; doi:10.1074/jbc.RA118.003613)
Supplement: Supporting Information [file supp_293_36_14178__index.html]

Transcription factor-7–like 2 (TCF7L2) gene acts downstream of the Lkb1/Stk11 kinase to control mTOR signaling, β cell growth, and insulin secretion — Lkb1 regulates Tcf7l2 action in β cells — Supporting Information 

# Transcription factor-7–like 2 (*TCF7L2*) gene acts downstream of the *Lkb1*/*Stk11* kinase to control mTOR signaling, β cell growth, and insulin secretion

## Supporting Information

- Supporting information - Supplemental tables and figures
