# Supplementary material for: Transcription factor-7–like 2 (TCF7L2) gene acts downstream of the Lkb1/Stk11 kinase to control mTOR signaling, β cell growth, and insulin secretion
Source: J Biol Chem. 2018 Jul 2;293(36):14178–89. doi: 10.1074/jbc.RA118.003613 (PMC6130960; doi:10.1074/jbc.RA118.003613)
Supplement: Supporting Information [file supp_RA118.003613_137644_1_supp_154882_p7df7p.pdf]

Supplemental Table S1

	Percentage of strain-specific SNP similarities<br><br>Comparing FVB vs C57BL6/J strain		Percentage of strain-specific SNP similarities<br><br>Comparing 129 vs C57BL6/J strain	
**Sample**	**FVB/NJ**	**C57BL6/J**	**129S1/SvImJ**	**C57BL6/J**
Control (group 1)	3.77%	96.23%	3.62%	96.38%
βLkb1-KO (group 1)	3.08%	96.92%	3.29%	96.71%
βLkb1-KO-Tcf7l2-het (group 1)	4.14%	95.86%	4.93%	95.07%
βLkb1-KO-Tcf7l2-het (group 2)	5.52%	94.48%	2.96%	97.04%
βLkb1-Tcf7l2-dKO (group 2)	7.19%	92.81%	3.95%	96.05%

Table S1: Confirmation of strain identity in the new mouse line.

Results were obtained from JAX® through SNP Genome Scanning services which determined the percentage of three different genetic backgrounds in mouse line. 304 strain-specific SNP were analyzed from genomic DNA extracted from ear notch (one mouse per genotype from one litter in each group). The percentage represents the number of identical SNP observed in the control animals from JAX (FVB and 129S1). FVB/NJ is compared to C57BL/6J and 129S1/SvImJ is compared to C57BL/6J.

Supplemental Table S2

Gene	Forward sequence	Reverse sequence
Axin-2	CCACCGTGGTTGGCTTGT	GAAGGACCTGAATCCGTTTTCA
GSK-3β	AGCCTTCAGCTTTTGGTAGCAT	GAGTTGCCACTACTGTGGTTACCTT
Ctnnb1	TCCCTGAGACGCTAGATGAGG	CGTTTAGCAGTTTTGTCAGCTC
β-actin	CGAGTCGCGTCCACCC	CATCCATGGCGAACTGGTG
Tcf7l2	CAAAACAGCTCCTCCGATTCC	CTTGGCCGCTTCTTCCAA
Lkb1	GGGCTTCCACCTGGTGCCAGCCTGT	GAGATGGGTACCAGGAGTTGGGGCT

Table S2: PCR primers sequences.

Supplemental Figures

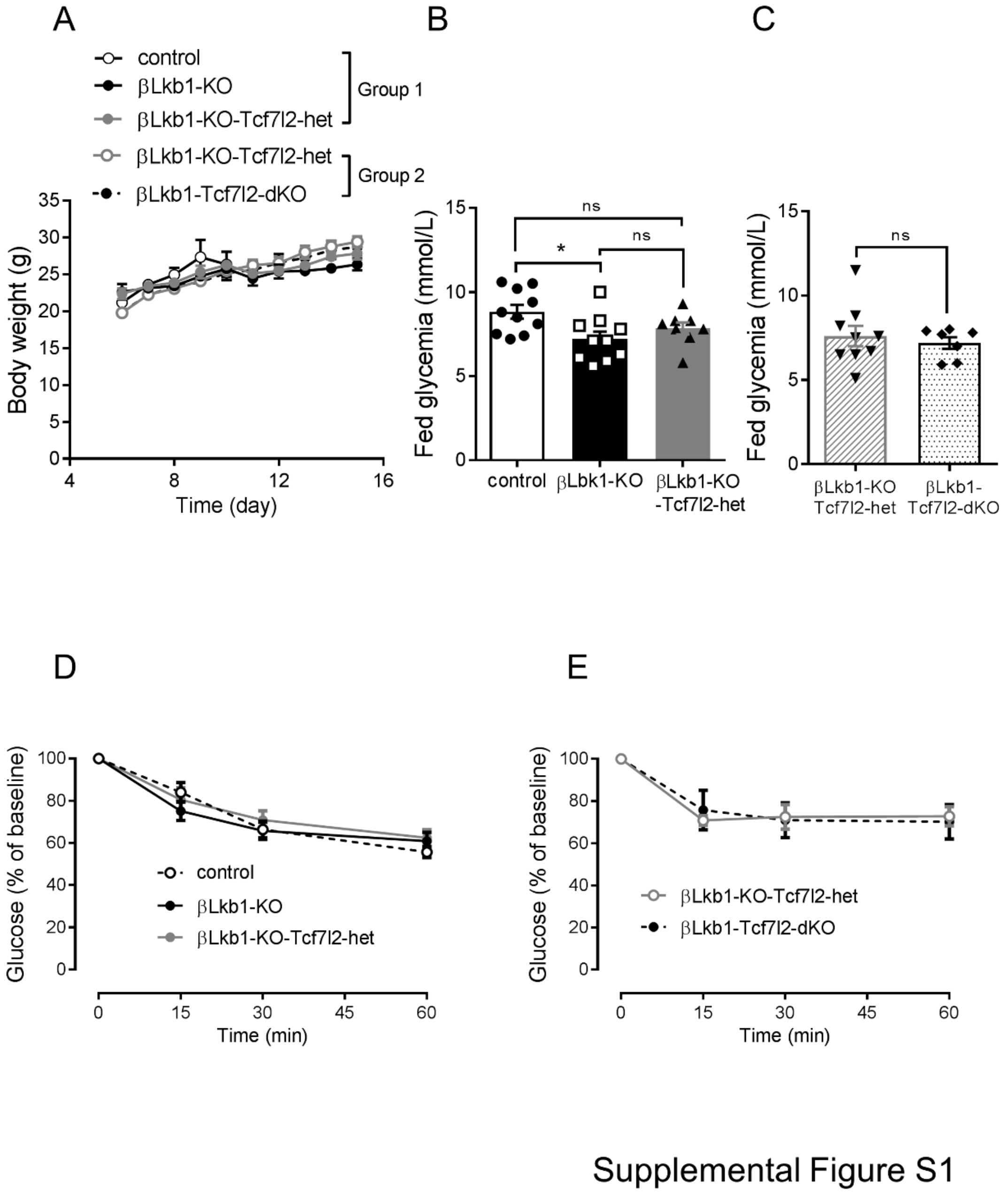

Figure S1: Deletion of one or two Tcf7l2 alleles do not affect insulin sensitivity or body weight and display not further decreased of fed glycemia in males.

A, B: Insulin sensitivity was measured *in vivo* after intraperitoneal injection of insulin (0.75 UI/kg). C: Body weight was measured every week from week 6 to 15. D, E: Fed glycemia was randomly measured from the tail vein n=6-8, *p<0.05 βLkb1-KO *vs* control.

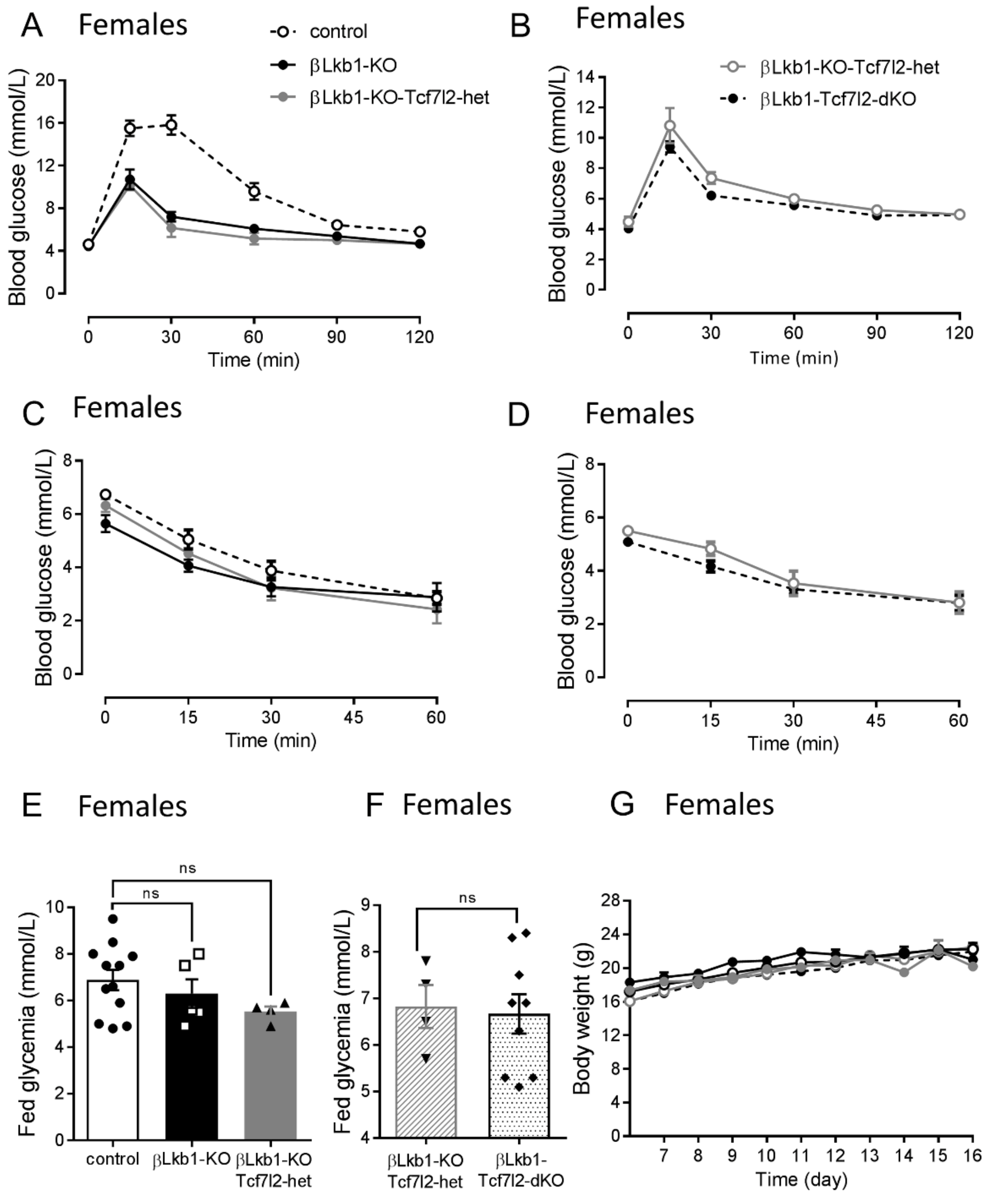

Supplemental Figure S2

Figure S2: Deletion of one or two Tcf7l2 alleles in females display unchanged glucose tolerance, insulin sensitivity, body weight and fed glycemia.

A, B: Oral glucose tolerance was measured after oral gavage of glucose (2 g/kg).

C, D: Insulin sensitivity was measured *in vivo* after intraperitoneal injection of insulin (0.75 UI/kg). E, F: Fed glycemia was randomly measured from the tail vein. G: Body weight was measured every week from week 6 to 16, n=8-10 mice/genotype.
